# Ultrasonographic and histopathological investigation of the effect of N-acetylcysteine on doxorubicin-induced ovarian and uterine toxicity in rats

**DOI:** 10.1186/s13048-024-01459-4

**Published:** 2024-06-28

**Authors:** Evren Üstüner, Ebru Yıldırım, Hasan Ceyhun Macun, Hüsamettin Ekici, Yaşar Şahin, Enes Güncüm, Tuğçe Anteplioğlu, Taha Burak Elifoğlu, Esra Bozkaya

**Affiliations:** 1 Faculty of Medicine, Department of Radiology, Ankara University, Ankara, Turkey; 2 Faculty of Veterinary Medicine, Department of Pharmacology and Toxicology, Kirikkale University, Kirikkale, Turkey; 3 Faculty of Veterinary Medicine, Department of Obstetrics and Gynecology, Kirikkale University, Kirikkale, Turkey; 4 Faculty of Veterinary Medicine, Department of Pathology, Kirikkale University, Kirikkale, Turkey; 5 Scientific and Technological Research Application and Research Center, Kirikkale University, Kirikkale, Türkiye

**Keywords:** Doxorubicin; N-acetylcysteine, Ovarian toxicity, Uterine toxicity, Rat, Ultrasound

## Abstract

**Background:**

This study aimed to investigate the mitigating effect of N-acetylcysteine (NAC) on doxorubicin (DOX)-induced ovarian and uterine toxicity in rats using laboratory tests, ultrasonographic (US) imaging, and histopathology analysis.

**Methods:**

Forty-eight rats were divided into six groups (*n* = 8) as follows: Group A (control) (0.5 mL saline administered intraperitoneally [IP]), Group B (a single 10 mg/kg dose of DOX administered IP on day 1), Group C (a single 10 mg/kg dose of DOX administered IP 24 h before sacrifice), Group D (100 mg/kg of NAC administered IP for 21 days), Group E ( a single 10 mg/kg dose of DOX administered IP on day 1 and 100 mg/kg of NAC administered IP for 21 days), and Group F (100 mg/kg of NAC administered IP for 21 days and a single 10 mg/kg dose of DOX administered IP 24 h before sacrifice). The ovaries were examined using B-mode US on days 1, 14, and 21, and the histopathological examinations of the ovaries and the uterus were undertaken after sacrifice on day 22.

**Results:**

Histomorphological analyses showed that ovarian weight decreased after DOX administration in Group B but not in Group E. US revealed a transient increase in ovarian size in Group B and E, reverting to baseline levels over time, as well as a progressive increase in peritoneal fluid in Groups B and E. Group B exhibited a significant decrease in the thickness of the endometrium and myometrium and uterine cornual length, which was not observed in Group E. Histopathological examination showed that DOX caused a decline in follicular count, especially in primordial, secondary, and Graafian follicles, and resulted in follicular atresia, predominantly in Group B. Destructive degeneration/necrosis and vascular changes were most prominently seen in the corpus luteum of Groups C and B. In NAC-treated rats (Groups E and F), although germ cell damage was present, atretic follicles and vascular changes, such as hyperemia and congestion, were reduced. The anti-müllerian hormone (AMH) level was the highest in Group F.

**Conclusions:**

NAC, an antioxidant, attenuated DOX-induced gonadotoxicity in rats.

## Introduction

Doxorubicin (DOX) is an anthracycline chemotherapeutic agent that has been incorporated into many treatment protocols for neoplasms such as gynecologic, urologic, and lymphatic system cancers [1]. The exact mechanism of action of DOX is complex. Doxorubicin inhibits topoisomerase II, destabilizes DNA, and leads to chromatin damage [2]. Another mechanism of DOX is its ability to generate free radicals, radical oxygen species (ROS), and oxidative stress that induce DNA and cell membrane damage [1]. Damage through apoptotic pathways, including caspase activation, inhibition of actin phosphorylation, cytoskeleton instability, and vascular toxicity, has also been reported [3, 4]. Although side effects of DOX can be seen in many organ systems, the main dose limiting effects are cardiotoxicity, hepatotoxicity, bone marrow suppression, and reproductive toxicity [2, 5].

The improved prognosis of neoplasms over time has paralleled an increase in young female survivors suffering from chemotherapy-induced gonadotoxicity. Notably, acute ovarian failure has been reported to reach 10%, whereas infertility rates are approximately 40%, progressively increasing with advancing age [6]. Amenorrhea subsequent to the acute administration of chemotherapeutics and early menopause have also been extensively documented [6, 7]. DOX has negative effects on female fertility, causing amenorrhea, primary ovarian insufficiency, premature menopause, infertility, small litter size, and increased genetic aberration risk [6–8]. The incidence of amenorrhea following DOX-containing protocols can range between 20% and 80%, depending on the patient’s age [8, 9]. Therefore, there is a pressing need for protective fertility measures and the development of new strategies to reduce the detrimental effects of DOX treatment on the reproductive system.

Oxidative stress and apoptosis are postulated as cellular processes implicated in the etiology of DOX-induced gonadotoxicity [2]. N-acetylcysteine (NAC) is a potent chelator, antioxidant, anti-inflammatory, mucolytic, and cytoprotective agent that can act as a strong hydroxyl radical scavenger, capable of cleaving disulfide bridges, increasing cysteine and glutathione levels, and modulating mitochondrial dysfunction and apoptosis [10, 11]. NAC can also alter the levels of neurotransmitters such as glutamate and dopamine and regulate intracellular signaling [11]. Possessing a well-documented safety profile, NAC is a safe, widely available drug, with rare occurrences of overdoses and side effects [10, 11]. These unique properties of NAC have positioned it as a subject of extensive research endeavors aimed at mitigating side effects and alleviating toxicity.

The aim of this study was to investigate the protective effect of NAC on DOX-induced ovarian and uterine toxicity in rats using laboratory tests, ultrasonographic (US) imaging, and histopathological analysis.

## Materials and methods

The study was conducted using female Wistar albino rats aged five to seven months, weighing 200–320 g. The rats were provided with a standard diet and access to water ad libitum throughout the experimental period. They were housed in polyethylene cages under a 12-hour light/dark cycle at a constant temperature of 24 ± 2 °C and a relative humidity of 50–60%. Prior to the experiments, the animals were kept in isolation for two weeks. The experiments were carried out at the experimental research center of Kirikkale University, Kirikkale, Turkiye. The study protocol adhered to the animal research guidelines and was approved by the Ethical Committee of the university (date: November 24, 2022, number: 2022/06–31). Forty-eight rats were randomly divided into six groups of eight animals each. For the experiments, DOX (Adriamycin 50 mg; Deva, Turkiye) and NAC (N-acetylcysteine-Asist 300 mg/3 ml; Bilim, Turkiye) were used. The experimental groups were designed as outlined in Table 1.

**Table 1:** Experimental groups

Group	Description
Group A (control)	Only 0.5 mL saline was injected intraperitoneally (IP).
Group B	A single 10 mg/kg dose of DOX was administered IP on the first day of the trial to examine the chronic effects of DOX.
Group C	A single 10 mg/kg dose of DOX was administered IP 24 h before sacrifice to examine the acute effects of DOX.
Group D	NAC was administered IP at a dose of 100 mg/kg for 21 days.
Group E	NAC was administered IP at a dose of 100 mg/kg for 21 days, and a single 10 mg/kg dose of DOX was administered IP on the first day of the trial to examine the efficacy of antioxidant NAC treatment on rat ovarian tissue exposed to DOX in the long term.
Group F	NAC was administered IP at a dose of 100 mg/kg for 21 days, and a single 10 mg/kg dose of DOX was administered IP 24 h before sacrifice In to examine the efficacy of pre-treatment with antioxidant NAC on rat ovarian tissue exposed to DOX acutely.

The dose and administration route for DOX [9, 12] and NAC were selected according to previous studies [13–15] that confirmed the in vivo and in vitro efficacy and safety of these agents. Assessment of ovarian activity entailed conducting vaginal smear and cytology examinations on rats within each experimental group every day over a three-day period to discern cyclic patterns or the presence of anestrus. These smears were compared repeatedly. Prior to vaginal smearing, the genital area of each rat was cleaned. The tip of a cotton swab applicator was moisturized with distilled water, gently inserted into the vagina, and rotated against the vaginal wall. The swab was then removed and rolled onto a glass slide immediately after retrieval. The smears were fixed in 85% ethanol for ten minutes and allowed to air dry. The smears were stained using Giemsa stain (Merck), following the manufacturer’s instructions, for a period of 40 min, after which they were washed with tap water. Following a second air drying process, the slides were examined under a microscope, and estrus stages were identified as described by Cora et al. [16].

The ovaries were evaluated by dynamic ultrasonography imaging using the B-mode at three distinct time points: 24 h after the beginning of the study (day 1), on day 14, and on day 21. These imaging sessions were scheduled to occur one hour subsequent to drug administration. An ultrasound device dedicated to veterinary ultrasound imaging was used with a dedicated 7.5-12-MHz linear broadband probe (SIUI V9, Shantou Institute of Ultrasonic Instruments [SIUI] Co. Ltd.). To visualize the ovaries, the small animal setting was chosen. The left abdominal sides of the rats were shaved, and ultrasound gel was generously applied as a coupling agent. The rats were held and restrained in the right lateral recumbent position on the table by a veterinarian, and the shaved left abdominal sides were exposed to US probe examination. Challenges encountered during this imaging process, such as the rapid breathing and mild wriggling movements of the rats, were overcome by completing the imaging process within a timeframe of one to two minutes.

The examination commenced by placing the probe transversely just below the ribs, a location chosen for its optimal visualization of abdominal structures. The kidney, being the most prominent and easily detected structure within the abdomen, presented as a bean-like, slightly echogenic structure. After localization of the kidney, the probe was maneuvered in a sagittal clockwise rotation to identify the caudal pole of the kidney. Ovarian identification was facilitated by their typical positioning within the fat pad, either medial or lateral to the lower pole of the kidney, presenting as oval or rectangular, hypoechoic structures. Care was taken to differentiate ovaries from intraabdominal lymph nodes, adrenal glands, uterine horns, and intestines, especially in the presence of ascites.

While examinations involving the use of probes with a frequency above 30 MHz allow for visualization of follicles within the ovary and enable measurement of vascularization changes through Doppler US, the current study employed probes with lower frequencies (7.5–12 MHz). Consequently, follicular structures and vascularization could not be clearly distinguished. Therefore, the assessment primarily focused on ovarian size and the general echogenicity of the ovaries. The length, width, and area of the ovaries were measured, and the presence and quantity of ascites in the abdomen and around the ovary were noted. The quantity of ascites in the peritoneum was graded as mild (I), moderate (II), and high (III) [17, 18].

The uterus is a V-shaped structure consisting of linear horns converging at the caudal abdominal midline. During US examination, these horns manifest as linear, tubular, elongated, hypoechoic, well-circumscribed structures of approximately 1 mm in size. However, differentiation of uterine horns from adjacent intestinal and muscular structures poses challenges, particularly when utilizing low-frequency probes. Consequently, US examination of the uterus was not included in the analysis due to the difficulty and time-consuming nature of the localization and measurement of the horns caused by their small size and echogenicity, as well as the presence of ascites in rats further impeding accurate differentiation of the horns from edematous intestines in rats in the presence of ascites [17].

The study lasted for 21 days. At the end of the experiment, on day 22, all rats were anesthetized using a combination of 10 mg/kg of xylazine (Alfazyne) and 90 mg/kg of ketamine (Ketalar), followed by the aspiration of blood by cardiac puncture. The serum was separated and kept at -20 °C for subsequent analyses. After an abdominal section, the ovaries and uterus of the rats were removed and dissected. The ovarian and uterus samples were also examined histopathologically. In addition, serum levels of anti-Müllerian hormone (AMH) were measured using an enzyme-linked immunosorbent assay kit (Elabscience, cat no: E-EL-R3022) according to the kit protocol, and the absorbance readings were recorded on the Microplate reader device (BIO-TEK EL X 800-Aotu strip washer: BIO SINGLE HAND X 50) at a wavelength of 450 nm.

### Histopathological analysis

Ovarian and uterine tissue samples were fixed in 10% buffered formalin and subsequently embedded in paraffin wax. Then, tissue sections measuring 4–5 μm were prepared and stained with hematoxylin and eosin. The slides were examined by light microscopy (Olympus BX51, Tokyo, Japan), and digital photomicrographs were taken.

For the assessment of ovarian histopathology, serial tissue sections were used to estimate the number of follicles. Sampling was conducted at regular intervals of every 10th section, resulting in a total of six sections being randomly selected from both the right and left ovaries. Ovarian follicles were categorized according to the classification proposed by Flaws et al. [19], as outlined in Table 2.

**Table 2:** Categorization of ovarian follicles

Categorization of follicles	Description
Primordial follicles	Follicles with an oocyte characterized by a single layer of fusiform-shaped granulosa cells
Unilaminar primary follicles	Follicles with an oocyte characterized by a single layer of cuboidal granulosa cells
Multilaminar primary follicles	Follicles with an oocyte characterized by two to three layers of cuboidal granulosa cells
Secondary follicles	Follicles with more than five layers of cuboidal granulosa cells and antral spaces between these cells
Graaf follicles	Follicles with the cumulus-oocyte complex and antral spaces
Atretic follicles	Follicles with a degenerated oocyte, disorganized granulosa cells, and apoptotic bodies in granulosa cells

Histomorphometrical analyses were performed to measure endometrial and myometrial thicknesses (µm) in three different fields for each histological sample using Image J [12].

### Statistical analysis

The statistical analysis was performed using IBM SPSS v. 26 (SPSS Inc., Chicago, IL, USA). All numerical data was expressed as mean ± standard deviation (SD) and frequency values, as appropriate. The distribution of the data was evaluated using the Shapiro-Wilk test and graphically. The one-way analysis of variance test was used for the comparison of three or more normally distributed groups, and the post hoc Bonferroni or Duncan test was used for pairwise comparisons. The comparison of non-parametric data between three or more groups was performed using the Kruskal-Wallis test. The Friedman test was conducted to evaluate the repeated measurements that did not show a normal distribution, while the repeated-measurestest was employed for those with a normal distribution, and the Bonferroni-Dunn test was used for pairwise comparisons. In the intragroup comparisons of variables with two follow-ups, the paired-samples t-test was used for those with a normal distribution and the Wilcoxon signed-rank test for those with a non-normal distribution. The Fisher-Freeman-Halton test was used to compare qualitative data. The tests were performed at a 95% confidence interval, and the significance level was based on *p* < 0.05.

## Results

Analysis of vaginal smear cytology showed that all animals were in a healthy estrous cycle, with no instances of acyclicity or anestrus observed among the subjects.

### US imaging

**Fig. 1:**
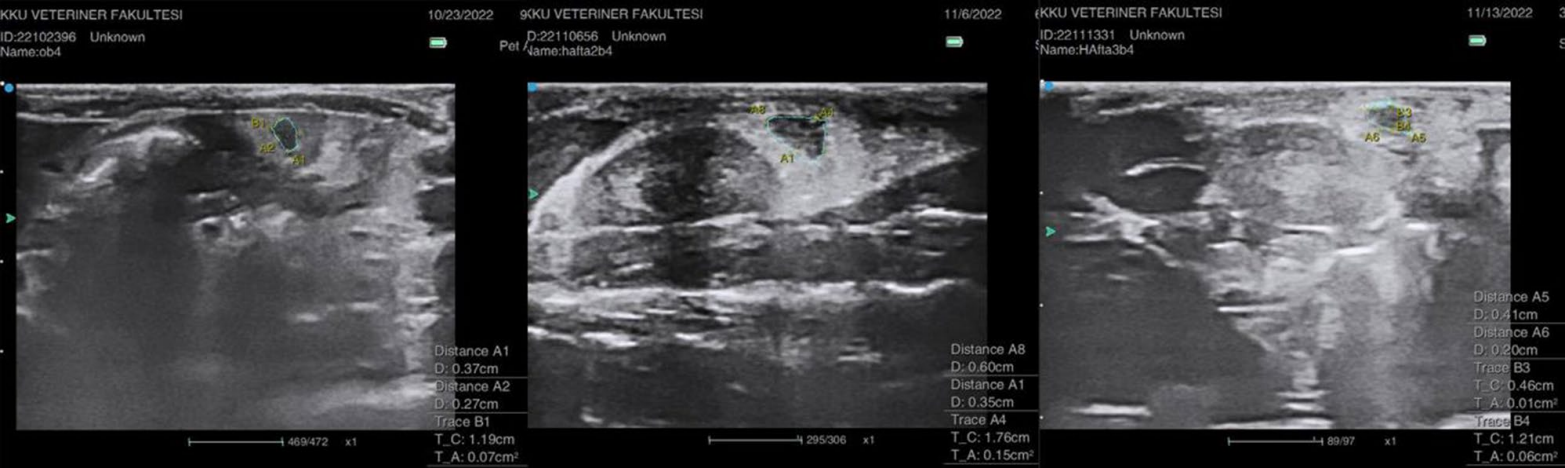
Ultrasound images of the ovaries of rat b4 from Group B on days 1, 14, and 21 of the study, indicating a gradual increase in peritoneal fluid

**Fig. 2:**
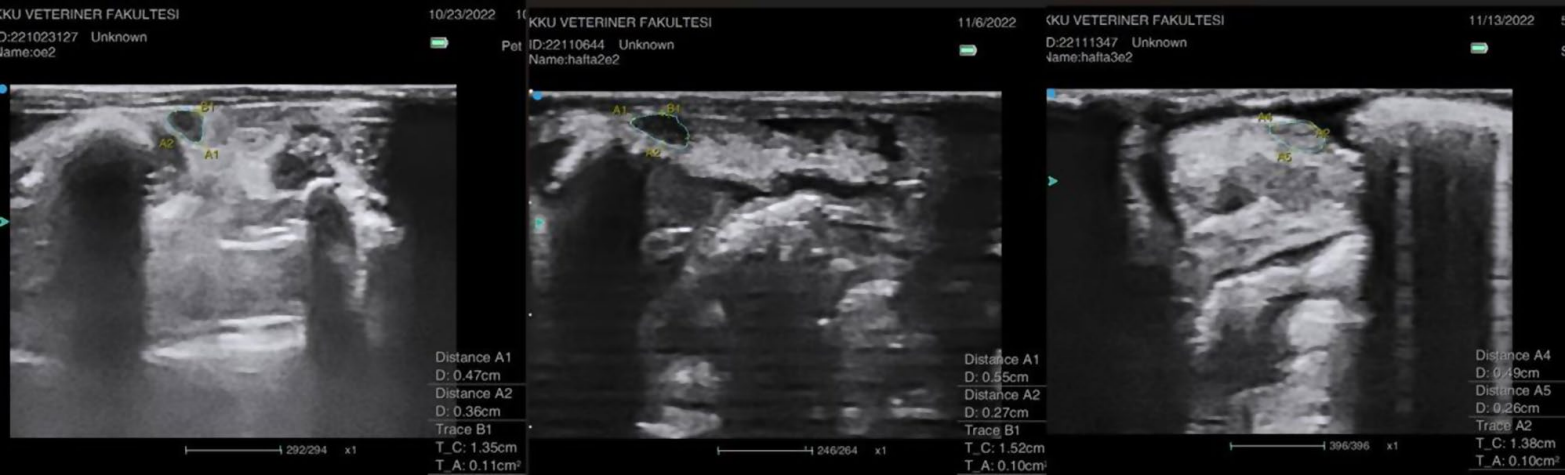
Ultrasound images of the ovaries of rat e2 from Group E on days 1, 14, and 21 of the study. TA denotes the area measurement. A gradual increase in peritoneal fluid is noted

There were no significant differences between the area measurements of the experimental groups on day 1 (*p* > 0.05). In Group B, ovarian area increased significantly from day 1 to 14, (*p* = 0.034), then significantly decreased from day 14 to 21 (*p* = 0.046) (Fig. 1). On day 14, a significant increase in ovarian area was detected in Group E compared to Group A (*p* = 0.021; *p* < 0.05) (Fig. 2). No significant size difference was noted in Group E on day 21 compared to Group A or day 1. On day 21, the ovarian size of Group C was significantly higher than that of Group A (*p* = 0.044; *p* < 0.05). The intergroup changes in size from day 1 to day 14 to day 21 were not significant in the remaining groups (Table 3). The overall trend was an increase in ovarian size in response to acute DOX administration, followed by a decrease in size over time.

**Table 3:** Assessment of ovarian area measurements among the groups using B-mode ultrasound

	Ovarian area measurements in cm^2^ (mean ± SD)				Intergroup differences *p*^b^		
Group	Day 1	Day 14	Day 21	*p* ^a^	Day 1 vs. 14	Day 1 vs. 21	Day 14 vs. 21
A	0.09 ± 0.003	0.07 ± 0.02	0.08 ± 0.02	0.250	0.401	1.000	1.000
B	0.07 ± 0.02	0.13 ± 0.04	0.08 ± 0.04	0.048*	0.034*	0.901	0.046*
C	0.10 ± 0.004	0.13 ± 0.05	0.14 ± 0.04	0.206	0.507	0.312	1.000
D	0.10 ± 0.02	0.11 ± 0.03	0.09 ± 0.02	0.236	1.000	0.782	0.401
E	0.12 ± 0.03	0.15 ± 0.06	0.09 ± 0.06	0.185	1.000	0.507	0.312
F	0.09 ± 0.03	0.28 ± 0.55	0.10 ± 0.03	0.670	1.000	1.000	1.000
*p* ^c^	0.120	0.012*	0.031*				

^a^Friedman test, ^b^Post hoc Bonferroni-Dunn test, ^c^Kruskal-Wallis test. *p < 0.05. *SD* standard deviation

Peritoneal fluid/ascites was not present in any of the experimental groups on day 1. However, fluid gradually increased, becoming detectable during the 14th - and 21st -day US examinations of Groups B and E (*p* = 0.001; *p* < 0.01). On day 14, there was a mild amount of fluid (Grade I) in Groups B and E compared to the remaining groups (*p* < 0.01). The amount of peritoneal fluid/ascites progressively increased over time, reaching significantly high levels (Grade III) in Groups B and E on day 21 (*p* < 0.01). In addition, in Group B, the increase in the amount of fluid from day 14 to day 21 was statistically significant (*p* = 0.023; *p* < 0.05). However, in Group E, the increase from day 14 to day 21 was not statistically significant (*p* > 0.05) (Table 4).

**Table 4:** Peritoneal fluid/ascites progression from day 14 to 21 by group

Group		A	B	C	D	E	F	*p* ^a^
Day 14	0	8 (100)	0 (0)	8 (100)	8 (100)	0 (0)	8 (100)	0.001*
	1	0 (0)	7 (87.5)	0 (0)	0 (0)	4 (50)	0 (0)	
	2	0 (0)	1 (12.5)	0 (0)	0 (0)	1 (12.5)	0 (0)	
	3	0 (0)	0 (0)	0 (0)	0 (0)	3 (37.5)	0 (0)	
Day 21	0	8 (0)	0 (0)	8 (0)	8 (0)	0 (0)	8 (0)	0.001*
	1	0 (0)	2 (25)	0 (0)	0 (0)	1 (12.5)	0 (0)	
	2	0 (0)	3 (37.5)	0 (0)	0 (0)	3 (37.5)	0 (0)	
	3	0 (0)	3 (37.5)	0 (0)	0 (0)	4 (50)	0 (0)	
*p* ^b^		1.000	0.023**	1.000	1.000	0.157	1.000	

^a^Fisher-Freeman-Halton test, ^b^Wilcoxon signed-rank test, *p < 0.01, **p < 0.05

### Histomorphometrical analysis of the ovaries

Histomorphometric evaluation showed that the right and left ovarian diameters were similar in all groups. A slight decrease in ovarian weight was noted following DOX use, which was statistically significant in Groups B, C, and F. This decrease was not present in Group E, which had received NAC for 21 days after a single dose of DOX on day 1 (Table 5).

**Table 5:** Ovarian weight and measurements

Group	Right ovary diameter (mm)	Left ovary diameter (mm)	Right ovary weight (g)	Left ovary weight (g)
A	0.53 ± 0.03	0.54 ± 0.04	0.08 ± 0.02^a^	0.09 ± 0.03^a^
B	0.50 ± 0.05	0.50 ± 0.06	0.06 ± 0.00^b^	0.06 ± 0.01^c^
C	0.51 ± 0.06	0.52 ± 0.10	0.07 ± 0.03^ab^	0.07 ± 0.02^bc^
D	0.51 ± 0.02	0.55 ± 0.05	0.07 ± 0.01^ab^	0.09 ± 0.02^ab^
E	0.53 ± 0.06	0.54 ± 0.07	0.09 ± 0.02^a^	0.09 ± 0.03^ab^
F	0.48 ± 0.06	0.48 ± 0.08	0.06 ± 0.01^b^	0.06 ± 0.01^c^
*p*	0.454	0.300	0.014*	0.004*

Statistical analysis was performed using one-way analysis of variance with the post hoc Duncan test. *p < 0.05. All values are given as the mean ± standard deviation for each group. Different letters within the same row indicate statistically significant differences

### Histomorphometrical analysis of the uterus and uterus layers

Uterine length and endometrial thickness decreased with DOX use, and this decrease was statistically significant in Groups B and F compared to Group A (controls) (Table 6). A statistically non-significant decrease in ovarian weight, along with a statistically significant decrease in myometrial thickness, was noted in Group B compared to Group A. Uterine length and weight and endometrium and myometrium thicknesses did not significantly differ between Group E and Group A (Table 6).

**Table 6:** Histomorphometric analysis of the uterus and uterus layers of all groups

Group	Right uterus horn length (mm)	Left uterus horn length (mm)	Uterus weight (g)	Thickness of endometrium (µm)	Thickness of myometrium (µm)
A	3.79 ± 0.53^ab^	3.86 ± 0.35^a^	0.46 ± 0.14	430.63 ± 90.10^a^	178.00 ± 40.37^a^
B	2.83 ± 0.27^dc^	2.94 ± 0.55^bc^	0.33 ± 0.06	255.00 ± 73.60^c^	110.00 ± 13.07^b^
C	3.43 ± 0.51^bc^	3.83 ± 1.20^a^	0.54 ± 0.23	373.50 ± 126.08^ab^	182.38 ± 62.84^a^
D	3.29 ± 0.80^bcd^	3.40 ± 0.51^ab^	0.53 ± 0.10	380.50 ± 103.26^ab^	204.00 ± 29.81^a^
E	4.34 ± 0.85^a^	3.63 ± 0.49^a^	0.53 ± 0.21	339.00 ± 38.41^abc^	175.13 ± 17.96^a^
F	2.71 ± 0.42^d^	2.70 ± 0.29^c^	0.45 ± 0.24	319.00 ± 42.21^bc^	166.63 ± 24.97^a^
*p*	< 0.0001*	0.002*	0.177	0.005*	< 0.0001*

Statistical analysis was performed using one-way analysis of variance with the post hoc Duncan test. *p < 0.05. All values are given as the mean ± standard deviation for each group. Different letters within the same row indicate statistically significant differences

### Histopathological analysis of the ovaries

**Fig. 3:**
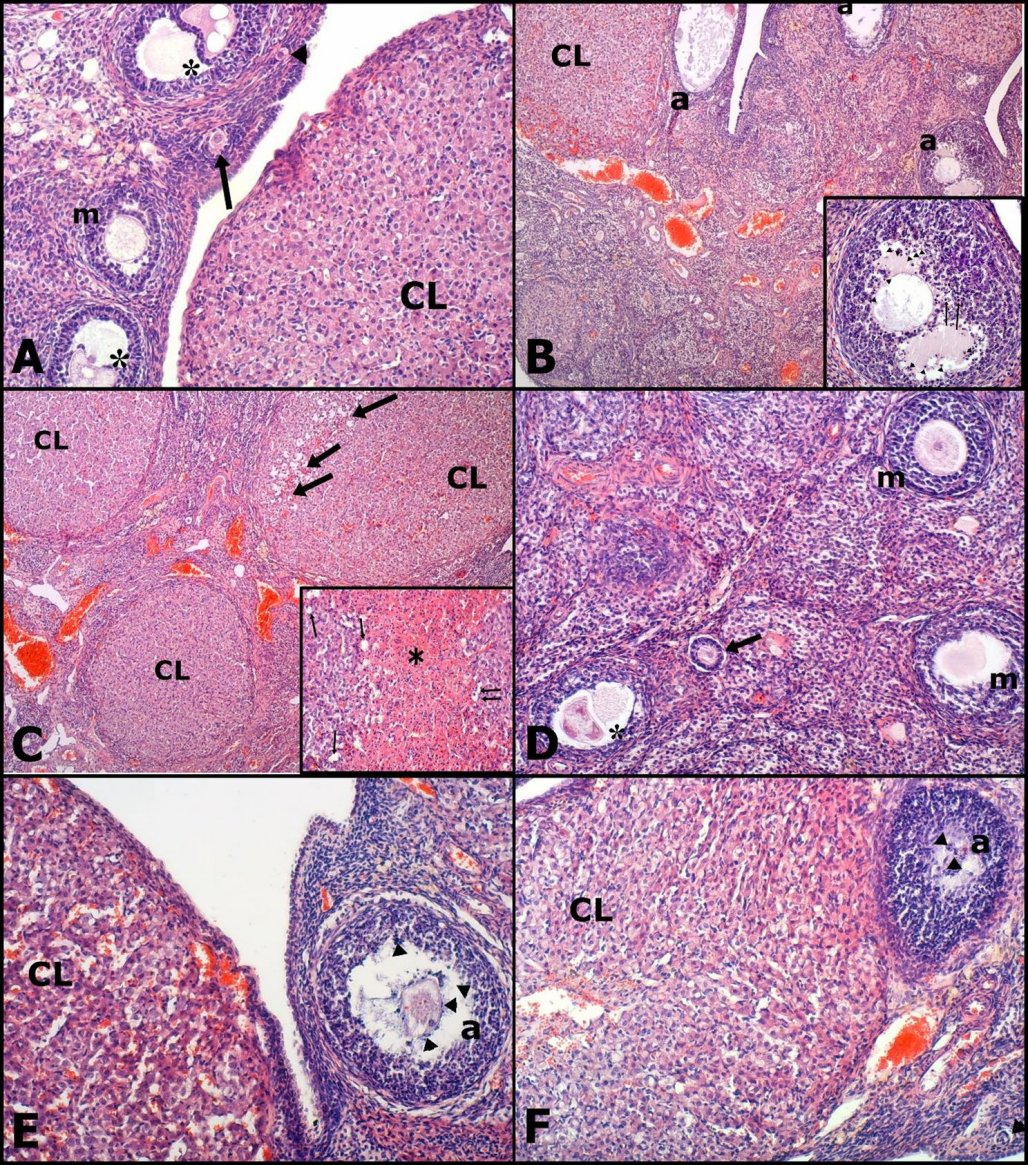
Histopathological sections of the ovaries. All sections stained with hematoxylin and eosin. **Group A**: Normal architecture of Primordial Follicle (arrowhead), Unilaminar Primary Follicle (arrow), Multilaminar Primary Follicle (m), Secondary Follicle (asterisk) and Corpus Luteum (CL). Scala bar: 320 μm. **Group B**: Mild hemorrhagia and vacuolation in Corpus Luteum (CL), congestion and Atretic Follicles (a). Scala bar: 110 μm. Inset panel: Apoptosis (arrowheads) and picnosis (arrows) in granulosa cells in atretic secondary follicle. Scala bar: 180 μm. **Group C**: Severe hemorrhagia and vacuolation (arrows) in Corpus Luteum (CL) and moderate to severe congestion with mild mononuclear cell infiltration. Scala bar: 110 μm. Inset panel: Vacuolar degeneration (arrows) and necrosis (asterisk) in center of CL. Scala bar: 180 μm. **Group D**: Normal architecture of Primordial Follicle (arrow), Multilaminar Primary Follicle (m), Secondary Follicle (asterisk) and mild edema in stratum. Scala bar: 320 μm. **Group E**: Mild hemorrhagia in Corpus Luteum (CL) and Atretic Follicles (a) with apoptotic bodies (arrowheads). Scala bar: 320 μm. **Group F**: Mild hemorrhagia and vacuolation in Corpus Luteum (CL), Atretic Follicles (a) with apoptotic bodies (arrowheads) and normal Primordial Follicle (arrow). Scala bar: 320 μm

The histopathological results of the ovaries were similar in Groups A and D, showing normal follicles in different stages of development, normal stromal tissue, and blood vessels (Fig. 3A, D). In Group B, there was a decrease in the number of healthy follicles and an increase in follicular atresia, with a reduction in primordial, secondary, and Graafian follicles. Atretic follicles were characterized by apoptotic and pyknotic granulosa cells and degenerated oocytes (Fig. 3B; Table 7). In Groups B and C, vacuolar degenerations and necrosis with hemorrhage in the corpus luteum were notable, being most prominent in Group C (Fig. 3B, C). In addition, vascular congestion and mononuclear cell infiltration were prominently observed in Groups B and C, with the latter demonstrating a higher predominance of these features (Fig. 3B, C).

**Table 7:** Number and distribution of ovarian follicles by group

Group	Primordial	Unilaminar	Multilaminar	Secondary	Graafian	Atretic
A	18.25 ± 3.58^a^	2.00 ± 1.41	3.25 ± 1.83	3.38 ± 2.00^a^	1.88 ± 1.96^a^	2.25 ± 1.49^bc^
B	9.5 ± 2.27^d^	2.00 ± 1.31	1.75 ± 1.28	1.38 ± 0.92^b^	0.25 ± 0.46^b^	5.25 ± 1.04^a^
C	11.5 ± 3.25^cd^	2.25 ± 1.39	2.13 ± 1.25	1.88 ± 1.46^b^	1.38 ± 1.06^ab^	3.13 ± 1.46^b^
D	16.13 ± 3.00^ab^	2.25 ± 1.04	3.38 ± 1.06	2.13 ± 1.25^ab^	2.25 ± 1.04^a^	1.13 ± 0.83^c^
E	13.00 ± 3.02^bc^	2.25 ± 1.04	2.38 ± 1.51	1.50 ± 0.76^b^	0.50 ± 0.53^b^	2.88 ± 1.64^b^
F	14.5 ± 2.93^bc^	1.88 ± 1.46	2.37 ± 1.06	1.00 ± 0.76^b^	1.38 ± 1.30^ab^	2.50 ± 1.20^bc^
*p*	< 0.0001*	0.984	0.138	0.011*	0.010*	< 0.0001*

Statistical analysis was performed using one-way analysis of variance with the post hoc Duncan test. *p < 0.05. All values are given as the mean ± standard deviation for the eight rats in each group. Different letters within the same row indicate statistically significant differences

Groups E and F exhibited luteal cell degeneration, which was characterized by a decreased number of ovarian follicles and moderate follicular atresia, congestion, and vacuolation, in comparison to Groups A and D. In addition, Groups E and F showed less follicular atresia, degeneration, and congestion compared to Groups B and C (Table 7; Fig. 3E and F).

### Histopathological analysis of the uterus

**Fig. 4:**
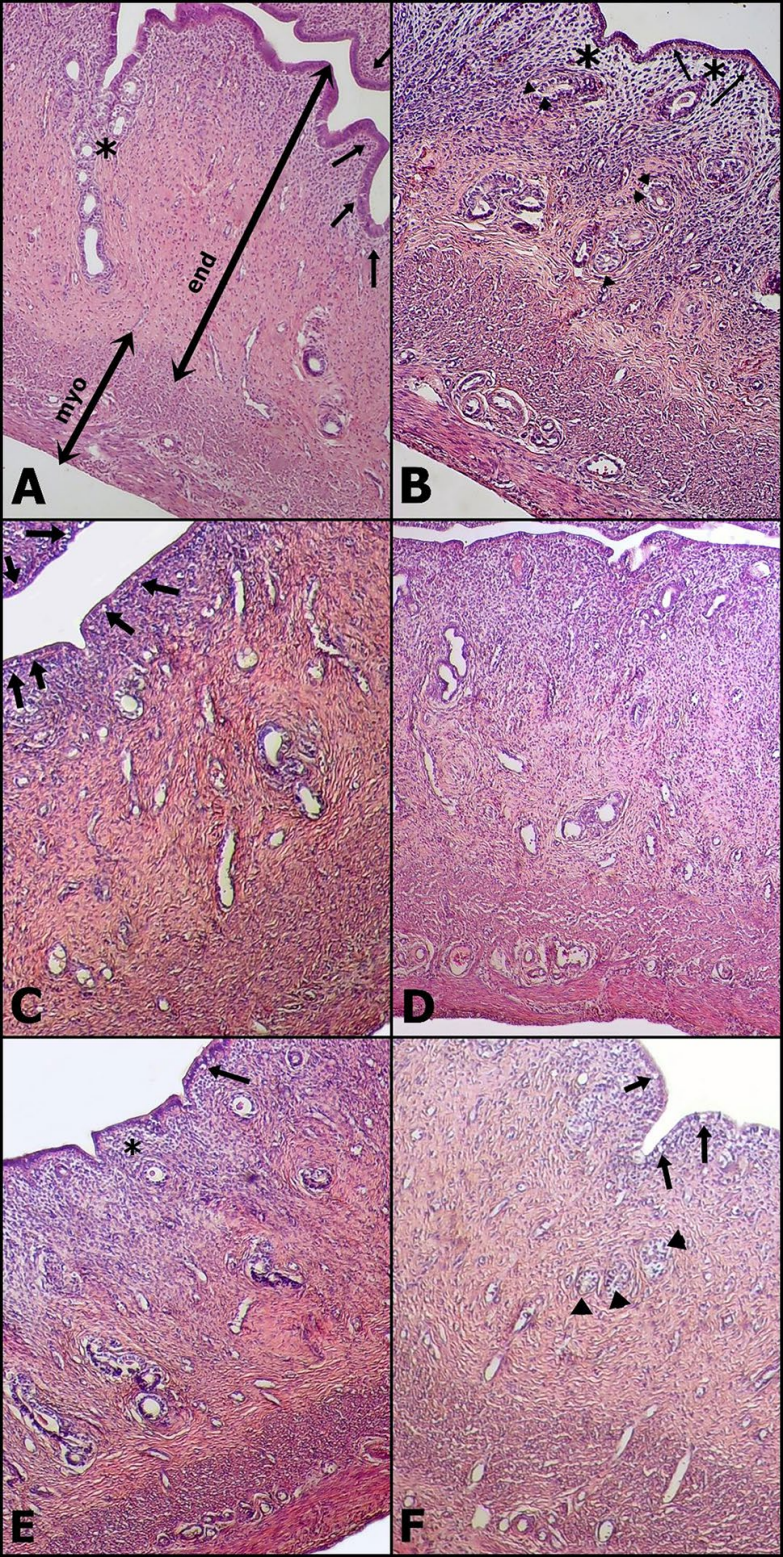
Longitudinal histopathological sections of the uteri. All sections stained with hematoxylin and eosin. Scala bar: 110 μm. **Group A**: Normal structure of uterine layers (Myo: Myometrium, End: Endometium), cuboidal endometrial epithelium (arrow) and endometrial glands (asterisk). **Group B**: Mild to moderate vacuolar degeneration of endometrial epithelium (arrows) and gland (arrowheads) and severe edema in endometrial stroma (asterisk). **Group C**: Moderate vacuolar degeneration of endometrial epithelium (arrows). **Group D**: Normal structure of uterus. **Group E**: Mild vacuolar degeneration of endometrial epithelium (arrow) and mild stromal edema (asterisk). **Group F**: Mild vacuolation of columnar (arrows) and glandular (arrowheads) epithelium

The microscopic examination of the uteri showed the presence of a normal columnar epithelium lining the lumen of the endometrium, along with glands embedded within the endometrial stroma, and a normal myometrium in Groups A and D (Fig. 4A, D). In contrast, Groups B and C exhibited vacuolation in the epithelial and glandular cells and marked stromal edema with tortuous endometrial glands and disorganized myometrium (Fig. 4B, C).

Groups E and F had similar structural architecture and histopathological lesions compared to Groups B and C, respectively; however, edema in the endometrial stroma was markedly reduced in both Group E and Group F (Fig. 4F, E).

These histopathological results show that DOX causes structural disorders in both the stromal and glandular structures of the uterus, especially in the endometrium, in the short and long term. Both histomorphometric and histological analysis revealed a decrease in stromal edema in both NAC-applied groups (E and F) compared to the groups that only received DOX (B and C).

### Laboratory analysis of AMH levels

AMH measurements showed statistically significant differences among the groups (*p* = 0.0001; *p* < 0.01). The blood AMH levels of Groups B, C, E, and F were significantly higher than those of Group A (*p* = 0.046, *p* = 0.048, *p* = 0.007, and *p* = 0.001, respectively) and Group D (*p* = 0.005, *p* = 0.019, *p* = 0.003, and *p* = 0.001, respectively). No significant difference was noted between Groups A and D (*p* > 0.05) (Table 8). While AMH levels did not statistically significantly differ between Groups B and E and between Groups C and F, they were numerically higher in Groups E and F. Remarkably, the AMH level in Group F was statistically significantly higher than those measured in Group B and Group E.

**Table 8:** Distribution of AMH levels (pg/ml) according to groups

Groups	AMH (pg/ml)
A (*n* = 8)	68.85 ± 25.39^a^
B (*n* = 8)	119.41 ± 56.14^b^
C (*n* = 5)	724.62 ± 387.77^bc^
D (*n* = 8)	49.87 ± 12.02^a^
E (*n* = 6)	468.17 ± 410.10^b^
F (*n* = 7)	1200.31 ± 430.31^c^
*p*	< 0.0001*

Statistical analysis was performed using the Kruskal-Wallis test with the post hoc Mann-Whitney U test. *p < 0.01. Different letters within the same row indicate statistically significant differences

## Discussion

This study aimed to investigate the detrimental effects of DOX administration on the reproductive system of rats and examine the effects of NAC, an antioxidant, on alleviating these effects. Histopathological examinations revealed a relative decline in ovarian weight in Group B at the end of the 21-day experimental period, which was consistent with long-term DOX-induced gonadotoxicity. There was also a decrease in ovarian size in groups that had received DOX, especially Group B; however, this decrease was not statistically different. The decrease in weight was consistent with the findings from previous studies conducted by Zhang et al. [7] and Ben-Aharon et al. [9]. Furthermore, in the current study, Group E, which received NAC for 21 days following a single dose of 10 mg/kg of DOX administration on the first day, did not show a decrease in weight or size, indicating the ameliorative effect of NAC.

In contrast to the findings of Ben-Aharon et al., who reported a slight decrease in mouse ovarian size during the first week using magnetic resonance imaging [9], our US imaging showed that ovarian size increased on the 14th day in Groups B and E and on the 21st day in Group C, which may be related to the acute edematous effects and acute vascular toxicity induced by DOX in rat ovaries. However, this increase in size in Groups B and E reverted to control levels in the long term (by the 21st day of the experiment), indicative of an overall trend of an initial increase in ovarian size in response to acute DOX administration, followed by a decrease in size over time. Histopathological examination further supported these findings, with acute changes such as edema and hemorrhage predominantly observed in Group C. Similarly, previous studies reported acute vascular toxicity, endothelial damage, and leaky vascular walls after acute DOX administration [3, 20]. In their magnetic resonance imaging study, Ben-Aharon et al. also noted significant periovarian edema in the acute phase in mice 24 h after DOX treatment, which was observed to decrease over time [9].

Structural disorders in both stromal and glandular structures in the uterus, especially in the endometrium, were noted following DOX administration. Uterine length, endometrial thickness, ovarian weight, and myometrial thickness all decreased with DOX use. Previous studies have primarily focused on ovarian damage induced by DOX, but our findings underscore the importance of uterine changes in contributing to reproductive toxicity. These observations are consistent with a study undertaken by Samare-Najaf et al., which attributed uterine changes to both the direct toxic effects of DOX and decreased ovarian function, resulting in reduced production of reproductive sex hormones [21]. The histomorphometric and histopathological examinations revealed less stromal edema and DOX-related changes in NAC-treated groups compared to the groups treated with DOX alone. In contrast, there were no significant changes in uterine size, weight, or endometrium and myometrium thicknesses in Group E that received NAC for 21 days following a single dose of DOX. Thus, the protective effect of NAC on the uterus appears to be more prominent with long-term use.

The development of peritoneal fluid/ascites was also a notable and concerning side effect of long-term DOX treatment, exhibiting a time-dependent incremental pattern. This finding was most notable in Groups B and E, gradually increasing over time. Similar observations were reported in Wistar rats by Wu et al., attributing this to DOX-induced cardiotoxicity leading to apoptosis of cardiomyocytes and endothelial cells, alongside vascular injury stemming from oxidative stress and endothelial barrier leakage [22]. Nevertheless, NAC demonstrated limited efficacy in mitigating this side effect.

According to the histopathological analyses, there was a significant decrease in follicular reserve following DOX administration, especially in primordial, secondary, and Graafian follicles over the long term. Concurrently, a significant increase was observed in atretic follicles and pyknotic granulosa cells. Acute changes were most pronounced in the corpus luteum, characterized by destructive degeneration/necrosis and vascular changes. NAC administration moderately reduced the damage caused by DOX, both in the short and long term. Although atretic follicles were observed, apoptosis was significantly less, and vascular changes such as hyperemia and congestion were reduced. Nonetheless, moderate follicular atresia, luteal cell degeneration, and a mild decrease in ovarian follicles were present. Previous studies have suggested that DOX primarily affects mitotically active follicles in rats, resulting in prominent apoptosis and a decrease in ovarian reserve [5, 9, 23–25]. Ben-Aharon et al. found a significant decrease in ovarian reserve, especially in secondary follicles [9], which is consistent with our findings. However, our study further noted a significant decrease in primordial follicles. Fabbri et al. investigated DOX and cisplatin in human ovarian cell cultures and found that apoptotic mechanisms were mainly responsible for the decrease in ovarian reserve [26].

The toxic effect of DOX is mainly on granulosa cells rather than oocytes within the growing follicles [27, 28]. AMH is secreted by granulosa cells and mediates ovarian reserve and follicular maturation [29]. Wang et al. suggested that DOX damaged mouse ovarian follicle reserve by causing primordial follicle atresia and overactivation of follicles [27]. High AMH levels may act as a suppressor of oocyte maturation, aiming to prevent this overactivation and maintain follicles in a dormant state, thus mitigating further primordial follicle loss, averting ovarian failure, and allowing rapid recovery in response to acute DOX administration [29]. In the current study, the relatively high AMH levels of Groups C and F after acute DOX administration support this hypothesis. Conversely, the relatively low AMH levels of Groups B and E compared to Groups C and F are likely related to granulosa cell loss and dysfunction, the decrease in mitotically active follicles, depletion of follicular reserve, and long-term apoptotic processes. The preventive effect of NAC on AMH levels was most pronounced in acute DOX administration, with no significant difference observed in the long term.

Many studies have delved into the deleterious effects of DOX on ovarian tissue. Both in vitro and in vivo studies on the ovarium and follicular cell cultures of humans and rats have demonstrated that DOX has detrimental effects on follicle reserve, growing follicles, oocyte maturation, steroid hormone production, DNA integrity, litter size, ovarian stroma, and vascularity [5, 9, 30]. The gonadotoxicity of DOX may involve multifaceted mechanisms affecting critical cellular targets, encompassing DNA damage, apoptosis, oxidative stress pathways, actin cytoskeleton instability, and vascular injury [4, 20, 24, 25, 30].

In a study by Zhang et al., DOX resulted in the highest decrease in ovarian volume and exerted the most toxicity in ovarian stroma through a decrease in ovarian reserve and ovarian fibrosis compared to other chemotherapeutics, namely cisplatin, cyclophosphamide, and paclitaxel. In the same study, chemotherapeutic damage to ovarian function was proposed to be through ROS-related oxidative damage, leading to mitochondrial dysfunction and ferroptosis-induced lipid peroxidation and apoptosis. The authors found that NAC alleviated cisplatin-induced ovarian toxicity by downregulating cellular ROS levels and enhancing antioxidative capacity [7]. Although this study parallels our findings indicating some protective effect of NAC on DOX-induced toxicity, different mechanisms are likely to be involved. In another study, Morgan et al. reported that cisplatin probably exerted ovarian damage through oocyte injury, whereas DOX induced this damage through granulosa cell injury, resulting in primordial and growing follicle loss [31]. This hypothesis is supported by experiments conducted in mouse ovarian cell cultures, where the tyrosine kinase inhibitor imatinib attenuated cisplatin-induced ovarian damage but had no effect on DOX-treated cells.

Wei et al. suggested that DOX-induced ROCK1-mediated inhibition of actin in the mouse embryonic fibroblast cytoskeleton led to impaired cell adhesion, activation of caspase pathways, and apoptosis [4]. While NAC inhibited DOX-induced caspase activation by 30% in mouse embryonic fibroblasts, ROCK1 deficiency elicited a stronger inhibitory response of 70–80% [32]. NAC was also more potent in suppressing H_2_O_2_-triggered caspase activity compared to DOX-regulated activation [4]. These findings may explain why NAC was partially effective in reducing DOX toxicity.

Brum et al. proposed a similar activity pathway for DOX [33]. The investigation involved an examination of the effect of DOX on cultured human ovarian cancer cells in vitro and revealed that pre-treatment with antioxidant NAC could enhance the efficacy of DOX in vivo by potentiating the ATM/p53 pathway, leading to cytoskeleton instability through ROS activity. In another study, de Lima et al. found that a high concentration of NAC was partially able to help impaired glucose uptake after DOX treatment in skeletal muscle cells [34]. The authors suggested that DOX interfered with AMPk signaling and the function of GLUT-4 receptors.

Farshid et al. observed the protective effects of histidine and NAC on sciatic neuropathy induced by DOX [35]. Similarly, Bulucu et al. found that NAC, selenium, and deferoxamine had alleviating effects on DOX-induced hepatocellular damage [36]. In a study by Doroshow et al., the effect of NAC on the cardiac toxicity of doxorubicin was examined in a mouse model, and the authors suggested that NAC might increase the sulfhydryl content in cardiac tissues and increase the chemotherapeutic effect of DOX [37]. Other research from the literature also indicates the preventive effect of NAC against cardiotoxicity and hepatotoxicity induced by DOX in rats [38, 39].

In this study, we found that the potent antioxidant NAC attenuated DOX-induced reproductive system toxicity over both short- and long-term periods. NAC was moderately successful in preventing loss of ovarian weight and size, uterine cornual length, and endometrial and myometrial thicknesses. It also moderately reduced ovarian edema, loss of follicular reserve, and apoptosis. However, it was not fully effective in preventing ascites and luteal cell degeneration. Mild follicular atresia and a decrease in ovarian follicles were present. Additionally, AMH levels were not as high as expected in the long term. This partial efficacy may stem from the heterogeneity in the mechanism of action of DOX. Lee et al. found that TP53 genetic alteration could affect antioxidant response, reporting that NAC supplementation enhanced the chemotherapeutic effect and reduced the adverse effects of DOX-induced ROS in TP53-altered cancers [40].

The administration of NAC for preventing gonadotoxicity subsequent to DOX exposure appears to be feasible, given its widespread use and accessibility. Previous studies on animal and human cell lines indicate that NAC works synergistically with DOX, enhancing its antitumoral efficacy and decreasing its side effects, particularly in TP53-altered cancers [7, 32–42].

In conclusion, our findings showed that NAC, an antioxidant, effectively attenuated DOX-induced gonadotoxicity in rats. NAC was especially successful in the prevention of apoptosis and granulosa cell damage but was not able to completely prevent follicle loss and ascites development. The response was somewhat partial and nowhere near complete, which is probably due to the biochemical heterogeneity in the mechanism of action of DOX, as well as the involvement of multiple pathways rather than the ROS system alone.

## Data Availability

No datasets were generated or analysed during the current study.
